# Characteristics associated with treatment seeking for smoking cessation among heavy-drinking research participants

**DOI:** 10.3389/fpsyt.2022.951364

**Published:** 2022-09-28

**Authors:** ReJoyce Green, Johnny Lin, Amanda K. Montoya, Mariel S. Bello, Erica N. Grodin, Howon Ryu, Diana Ho, Adam M. Leventhal, Lara A. Ray

**Affiliations:** ^1^Department of Psychology, University of California, Los Angeles, Los Angeles, CA, United States; ^2^Institute for Digital Research and Education, University of California, Los Angeles, Los Angeles, CA, United States; ^3^Institute for Addiction Science, University of Southern California, Los Angeles, CA, United States; ^4^Department of Preventive Medicine, University of Southern California, Los Angeles, CA, United States; ^5^Department of Psychiatry and Biobehavioral Sciences, University of California, Los Angeles, Los Angeles, CA, United States

**Keywords:** smoking cessation, random forest, treatment-seeking status, alcohol use severity, heavy drinking smokers

## Abstract

**Objective:**

Treatment seeking for smoking cessation has tremendous clinical implications with the potential to reduce tobacco-related morbidity and mortality. The present study seeks to elucidate clinical variables that distinguish treatment seeking versus non-treatment seeking status for smoking cessation in a large sample of heavy drinking smokers using data-driven methods.

**Materials and methods:**

This secondary data analysis examines *n* = 911 (*n* = 267 female) individuals who were daily smokers and heavy drinkers (≥ 7 drinks per week for women, ≥ 14 for men) that were enrolled in either a treatment-seeking study (*N* = 450) or a non-treatment seeking study (*N* = 461) using identical pharmacotherapies. Participants completed measures of demographics, alcohol and cigarette use, alcohol craving, the Barratt Impulsiveness Scale (BIS-11), and the Wisconsin Inventory of Smoking Dependence Motives (WISDM-68). These measures were used in a random forest model to identify predictors of treatment seeking status.

**Results:**

The top variables of importance in identifying treatment seeking status were: age, drinks per drinking day, cigarettes per smoking day, BIS-11 cognitive impulsivity, WISDM social environmental goads, WISDM loss of control, WISDM craving, and WISDM tolerance. Age and drinks per drinking day were two of the most robust predictors, followed by measures of nicotine craving and tolerance.

**Conclusion:**

Individuals who are daily smokers and consume more drinks per drinking day are less likely to belong to the smoking cessationtreatment-seeking group. Targeting heavy drinking smokers, particularly younger individuals, may be necessary to engage this group in smoking cessation efforts and to reduce the burden of disease of nicotine dependence earlier in the lifespan.

## Introduction

While cigarette use has declined over the last decade (1, 2), it remains a significant public health concern in the United States. The 2019 National Health Interview Survey (NHIS) found that approximately 21% of adults surveyed reported using a tobacco product, with 14% using cigarettes making it the most frequently used combustible product (3). One of the most used substances alongside cigarettes was alcohol. Cigarette smoking was twice as common among individuals twelve years and older with an alcohol use disorder (AUD) (37.8%) versus without (16.3%) in 2016 (4). Additionally, AUD was positively associated with meeting diagnostic criteria for any degree of severity for 12-month nicotine use disorder (NUD) (5). The co-use of cigarettes and alcohol in turn, results in an array of negative health consequences including increased risks of cancers and cardiovascular diseases (6, 7). Controlled laboratory studies have provided support for a strong bidirectional relationship between cigarettes and alcohol co-use. Both substances have been shown to increase craving and self-administration of the other substance (8). Therefore, studies have sought to elucidate the ways in which the use of alcohol and tobacco impacts one another.

The treatment implications of alcohol and cigarettes co-use are significant. Smokers engaging in a quit attempt are more than four times more likely to have a smoking lapse if they consume alcohol (9, 10). The effects of alcohol on smoking cessation ultimately render heavy drinking smokers more likely to experience poorer treatment outcomes compared to those who are not heavy drinkers (11–13). An examination of treatment preferences among heavy drinking smokers found a greater desire to quit smoking than drinking (14). Smokers with AUD endorsed withdrawal-related barriers to smoking cessation including feeling anxious or irritable, and concern related to experiencing unbearable cravings for cigarettes (15). Interestingly, the total number of perceived barriers was not related to severity of alcohol use, but was related to smoking history, expected effects of smoking, and temptation to smoke (15). Given these outcomes, heavy drinking smokers may be less likely to seek treatment due to difficulties in abstaining from one or both substances.

There is evidence on the prevalence and correlates of treatment seeking in separate literatures for AUD and NUD. Only 24.1% of individuals with lifetime AUD receive treatment (16), with an estimated gap of 8 years between the onset of an AUD and first seeking treatment (16). An examination of the natural progression of smoking quit attempts found most smokers make multiple shifts between smoking, reduction, and abstinence (11). Non-treatment seekers for AUD were more ethnically diverse, not married, lower education, and not working full-time (17). Specific to smoking, lack of knowledge on effective cessation methods and underestimating the benefits of various cessation strategies may serve as a barrier to seeking assistance for smoking cessation (18). Individual difference characteristics and mental health factors have also been associated with treatment seeking behavior for smoking cessation. Travaglini et al. (19) examined a sample of veterans who were regular cigarette smokers with a serious mental illness and found marital status, previous engagement with group smoking cessation services, and greater severity of positive psychotic symptoms were predictive of engaging in group treatment services for smoking cessation (19). However, little research has addressed the predictors of treatment seeking among smokers who are also heavy drinkers. To that end, one approach to parsimoniously screen and analyze the individual and interactive effects of numerous predictors on treatment seeking is the application of data driven methods. Lee and colleagues (20) aimed to identify what factors determined whether an individual with an AUD seeks treatment and found quantity and quality of alcohol use, drinking-related psychological problems, substance dependence, in addition to depression, race, BMI, and IQ as key measures predicting treatment seeking (20). No study has leveraged such data driven methods to predict treatment seeking for smoking cessation among populations of heavy drinkers.

The present study uses data driven methods (i.e., machine learning) to examine a host of clinical factors that distinguish treatment seeking versus non-treatment seeking status for smoking cessation in a large sample of heavy drinking smokers. By further understanding what clinical factors distinguish treatment versus non-treatment seeking for smoking cessation, we can identify potential intervention targets. Given the robust effects of alcohol on smoking, we also aim to examine how indicators of alcohol use may impact treatment seeking status for smoking cessation. Given emergent research demonstrating the utility of a machine learning framework in the treatment of substance use disorders (21), we analyze the data using a random forest model, a robust application of the machine learning data-driven approach.

## Materials and methods

### Participants and procedures

Participants consisted of non-treatment seeking and treatment-seeking heavy drinking smokers recruited from the larger Los Angeles community. Participants in the present study were pooled from 2 larger studies, a human laboratory study examining the effects of varenicline and low-dose naltrexone on subjective responses to cigarette and alcohol use (22) and a randomized clinical trial (ClinicalTrials.gov identifier: NCT02698215) comparing varenicline alone versus varenicline plus naltrexone for those who were treatment seeking, which was defined as an interest in smoking cessation and a desire to reduce or quit drinking (23). For the treatment seeking sample, participants were informed during the consent process that they would be required to set a smoking quit date as part of the active medication phase of the study. More detail on study procedures can be found elsewhere (23). Data for the present analyses was pulled from the initial screening visits for both studies. Both studies were approved by the Institutional Review Board of the University of California, Los Angeles. The research complies with the Declaration of Helsinki.

Inclusion criteria for participants included the present analyses was the following: (1) age between 21 and 55 years; (2) smoke 5 or more cigarettes per day; (3) meet National Institute on Alcohol Abuse and Alcoholism (NIAAA) heavy drinking guidelines, which for men consisted of 14 or more drinks per week, or 5 or more drinks per occasion at least once per month over the past year, and for women, 7 or more drinks per week, or 4 or more drinks per occasion at least once per month over the past year. Exclusion criteria included a lifetime history of bipolar disorders, psychotic disorders, or major depression with suicidal ideation. Participants completed a telephone screening prior to visiting the laboratory for an in-person screening visit. At the in-person screening visit, participants were required to have a breath alcohol concentration (BrAC) of 0.000 g/dl and test negative for all drugs, except cannabis, on urine drug test.

### Measures

The following individual difference measures were collected during the in-person screening visit: (a) demographics questionnaire; (b) Timeline Follow-Back [TLFB, (24)]; to gather standardized information regarding the number of cigarettes and standard alcoholic drinks consumed over the past 30 days; (c) Fagerström Test for Nicotine Dependence [FTND, (25)]; a self-report indicator of severity of nicotine dependence; (d) Wisconsin Inventory of Smoking Dependence Motives [WISDM-68, (26)]; to measure smoking motives; (e) Smoking History Questionnaire to assess previous smoking behavior; (f) Penn Alcohol Craving Scale [PACS, (27)]; to assess for intensity of alcohol craving; and (g) Barratt Impulsiveness Scale [BIS-11, (28)]; to assess for dimensions of impulsivity. These measures were selected based on existing literature noting important predictors of smoking cessation in clinical practice (29).

### Data analysis plan

All analyses were conducted in R 4.0.3. A set of 1000 random forest classification trees using the randomForest package was used to discover contributing factors to treatment seeking (30). Random forest is an extension of the single classification tree, where (a) each sample is randomly sampled with replacement and refit 1000 times (bootstrapped), and (b) a subset of variables is chosen for each bootstrapped tree. Random forest models have natural cross-validation since about 1/3 of participants are not selected in each bootstrap sample and considered “out of bag” (OOB). The available 2/3 of the data that are “in bag” serves as the training data and the remaining 1/3 out of bag sample is used to test the model found from training.

To limit the number of variables considered, 25 variables were included in the random forest. Cigarette use measures included nicotine dependence as indicated by total score on the FTND, cigarettes per smoking day (CPSD) gathered from the TLFB, and the following 13 smoking motives from the WISDM-68: affiliative attachment, automaticity, behavioral choice-amelioration, craving, cue exposure-associative processes, loss of control, negative reinforcement, positive reinforcement, social-environmental goads, taste and sensory properties, tolerance, and weight control. From the Smoking History Questionnaire, age of first cigarette, binary indicator of any quit attempt over the last year, and number of quit attempts over the last year were also included in the random forest. For the binary indicator of quit attempt over the last year, participants were able to indicate the date and duration of their two most recent quit attempts over the last year.

Alcohol use measures included alcohol craving as indicated by the PACS and drinks per drinking day (DPDD) derived from the TLFB. Mood related measures included the 2 factor scores from the BIS-11, cognitive impulsivity (or constraint) and behavioral impulsivity (or impulsivity) (31). The cognitive impulsivity score reflects constructs such as attentional control and planning, whereas the behavioral impulsivity score captures constructs such as acting impulsively and changing jobs (31). Lastly, three demographics characteristics included were age, sex, and race. Race was coded into three categories of White, Black, and All Other Races. This coding was due to small sample sizes for races outside of White and Black and was entered into the model as a single factor variable in R.

For each of 1000 bootstrapped samples, the random forest selects a subset of the 25 variables considered above. The recommended subset is the square root of the total number of variables (30). Since we considered 25 variables, a random subset of 5 of these variables were used in each bootstrapped tree. After fitting 1000 bootstrapped trees, the out of bag (OOB) error rate is generated as a measure of cross-validation. The lower the OOB error rate, the better the model is able to predict treatment seeking in the test sample. To assess the relative importance of each of the 25 variables, we calculated the Mean Decrease in Accuracy (MDA) and the Gini Index. For MDA, the more reduction in mean OOB prediction error, the more important the variable. The Gini index is an indicator of variable importance based on impurity reduction of the splits in the random forest model. Since the Gini and MDA produce a different set of importance rankings, we reported the union of the top 5 variables from both indexes to ensure the robustness of the findings.

**FIGURE 1:**
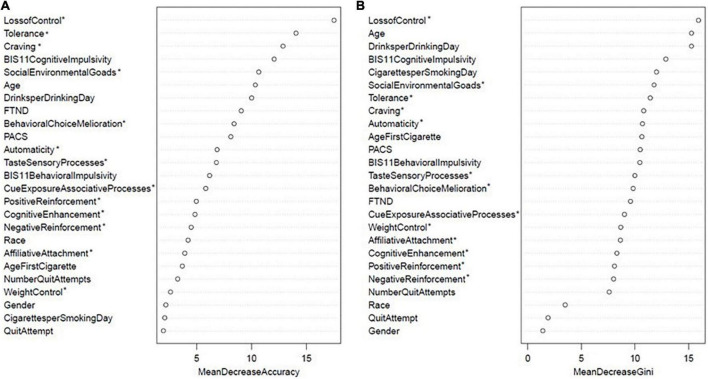
Random forest variable importance: **(A)** Mean decrease accuracy and **(B)** mean decrease gini scores for one seed. Variables with an asterisk indicate 13 subscales of the WISDM-68.

Figure 1 depicts the variable importance plot; each variable in a row can be interpreted as the standardized decrease in the accuracy or purity of the model had the variable been *excluded*. The OOB error is compared to the OOB error after permuting the variable under consideration. In other words, instead of removing the variable, the algorithm permutes the order of the variable down the observations so that the variable becomes independent of the outcome. The difference between the two errors is then averaged over all trees, and normalized by the standard deviation of the differences (32). Participants with missing data on the predictors (not the dependent variable) were multiply imputed using the rfImpute function. The same random forest model with a different seed of the multiple imputation was run with nine additional seeds in order to discover a pattern to generate multiple sets of variable importance rankings. For each seed, a rank of the variable importance from 1 to 25 was assigned separately for MDA and Gini. Of note, the variable importance plot in Figure 1 reflects one seed and is not an average across all seeds. We then selected the variables that were ranked in the top 5 across all ten seeds as our final set of variables with the largest impact on model accuracy. Between MDA and Gini, eight variables were chosen, and partial dependence plots were generated to assess the marginal effect each of these variables had on the log odds of treatment-seeking.

## Results

### Sample characteristics

Following this exclusion criteria, sample characteristics for both treatment seeking samples are summarized in Table 1. There was a total of 911 participants, 461 recruited as part of the non-treatment seeking sample and 450 recruited as part of the treatment-seeking sample. On average participants in the non-treatment seeking sample were approximately 7 years younger than participants in the treatment seeking sample. Participants reported similar cigarettes per smoking day in the non-treatment (sample mean ± sample standard deviation: 14.03 ± 7.64) and treatment seeking groups (12.48 ± 7.42). Participants also reported similar drinks per drinking day in the non-treatment (6.69 ± 3.74) and treatment seeking groups (5.75 ± 3.77).

**TABLE 1:** Sample characteristics.

Variable*	Non-treatment seeking (*n* = 461)		Treatment seeking (*n* = 450)	
	Mean	SD	Mean	SD
**Demographics**				
Age	36.10	10.60	43.25	12.32
Gender	N	%	N	%
Female	120	28.57	147	32.67
Male	300	71.43	303	67.33
Race				
White	177	42.45	139	30.89
Black or African-American	177	42.45	240	53.33
Another Race	63	15.11	71	15.78
**Cigarette use**	N	%	N	%
Quit attempt in last year^a^	209	45.34	255	56.67
Number of 24-hr quit attempts in last year	2.63	4.41	7.50	26.39
Cigarettes Per Smoking Day^b^	14.03	7.64	12.48	7.42
FTND^c^	4.42	2.28	4.70	2.21
Age of first cigarette	15.49	4.46	17.10	9.65
**Alcohol use**				
Drinks per Drinking Day^b^	6.69	3.74	5.75	3.77
PACS^d^	12.44	6.81	12.01	7.08
**Mood**	Mean	SD	Mean	SD
BIS-11 – Cognitive Impulsivity^e^	13.50	3.99	12.68	3.85
BIS-11 – Behavioral Impulsivity^e^	13.82	3.88	12.70	3.70

*There is some missing data across variables.

^a^Reflects the number and percentage who answered “yes” to this question.

^b^Assessed by TimeLine Follow-Back (TLFB) interview for the past 30 days.

^c^FTND = Fagerström Test for Nicotine Dependence.

^d^PACS = Penn Alcohol Craving Scale.

^e^BIS-11 = Barratt Impulsiveness Scale.

### Random forest model

For the particular seed displayed in Figure 1, the OOB error rate was 32.3%. This means that using the “in bag” bootstrap sample to train the model, the overall accuracy of the random forest model to predict the outcome in “out of bag” sample was 67.7%. The top eight variables from MDA in descending order were WISDM loss of control, WISDM tolerance, WISDM craving, BIS-11 cognitive impulsivity, WISDM social environmental goads, age, drinks per drinking day, and FTND at 17.5, 14.1, 12.9, 12.1, 10.7, 10.4, 10, 9.1, respectively (Figure 1). All other MDA scores were below 9. The top eight variables from Gini in descending order were WISDM loss of control, age, drinks per drinking day, BIS-11 cognitive impulsivity, cigarettes per smoking day, WISDM social environmental goads, WISDM tolerance, WISDM craving at 15.9, 15.3, 15.3, 12.9, 12.0, 11.8, 11.4, 10.8, respectively (Figure 1).

As discussed in the *Data Analysis Plan*, a total of 10 seeds of the same random forest model each with a different multiple imputation were run to assess the consistency in the rankings of top eight variables. Consistency was assessed by which variables appeared most frequently (the mode) in the top 5 within MDA and the top 5 within Gini. The average OOB error rate for the random forest model across ten seeds was 31.3%. This means that using the “in bag” bootstrap samples to train the model, the average accuracy of the random forest model to predict the outcome across all ten seeds was 68.7%. More specifically, the average marginal prediction accuracy of non-treatment was 87.2% whereas the average accuracy of treatment was only 31.3%, suggesting that the model was better at predicting non-treatment seeking than treatment seeking. A combination of the top 5 variables within MDA and top 5 within Gini metric resulted in the following top 8 variables of importance: age, drinks per drinking day, cigarettes per smoking day, BIS-11 cognitive impulsivity, WISDM social environmental goads, WISDM loss of control, WISDM craving, and WISDM tolerance. Across our 2 measures of relative importance, there was a general trend across the 10 imputations in which MDA metric often indicated WISDM subscales and BIS-11 cognitive impulsivity as top predictors, whereas Gini often indicated age and drinks per drinking day among the top predictors.

**FIGURE 2:**
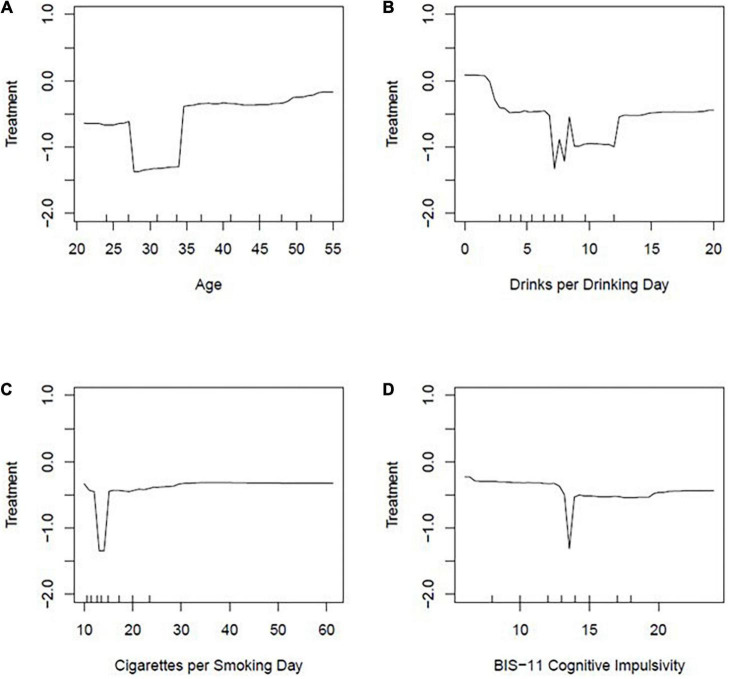
Partial dependence plots: **(A)** Age, **(B)** drinks per drinking day, **(C)** cigarettes per smoking day, **(D)** BIS-11 cognitive impulsivity. Partial dependent plots are displayed to show the effect of 4 variables (age, drinks per drinking day, cigarettes per smoking day, BIS-11 cognitive impulsivity) on the log-odds of treatment seeking status.

**FIGURE 3:**
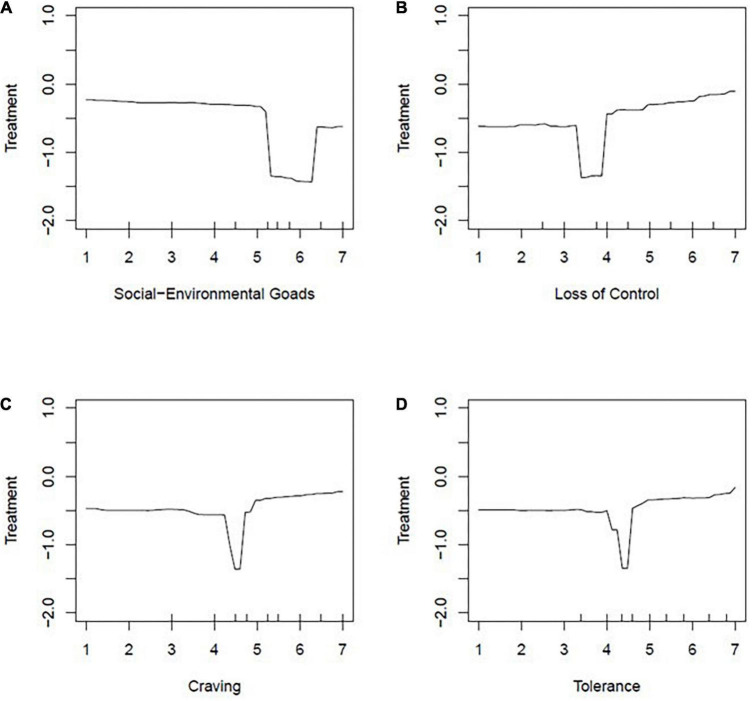
Partial dependence plots: Four of the WISDM-68 subscales: **(A)** Social environmental goads, **(B)** loss of control, **(C)** craving, **(D)** tolerance. Partial dependent plots are displayed to show the effect of 4 WISDM-Subscales (social environmental goads, loss of control, craving, tolerance) on the log-odds of treatment seeking status.

Although MDA and Gini assess relative importance, they do not indicate the direction of these impacts. Partial dependence plots allow us to individually assess the effect of each of these top eight variables on the log-odds of treatment seeking (Figures 2, 3). A note of caution is that since these models are predictive models, we make no assumptions about the inference or *p*-values associated with these effects. A value of zero in these partial dependence plots indicates a 50% chance of being categorized as treatment seeking and 50% of being categorized as non-treatment seeking. Across all top eight variables, none of the log odds of treatment-seeking were above zero. This suggest the effects were relatively small and that these eight variables did not do better than chance at predicting treatment-seeking. This is consistent with our findings that the average prediction accuracy of treatment-seeking was only 31.3%. Nevertheless, in the following paragraph, we qualitatively describe some notable trends. For the variables presented in Figure 2, there was a general trend with older age being associated with increased log odds of seeking treatment relative to those that were younger in our sample. When examining drinks per drinking day, the log odds of treatment seeking decreased as drinks per drinking day increased. For cigarettes per smoking day, the log odds of treatment seeking were relatively stable as a negative value regardless of smoking intensity. This result suggests that across the range of cigarettes per smoking day in our sample, participants were less likely to seek treatment. A similar pattern was seen for BIS-11 cognitive impulsivity scores such that there was a relatively stable negative log odds value of seeking treatment across the range of cognitive impulsivity scores. Thus, regardless of severity of cognitive impulsivity, participants were less likely to seek treatment.

Of the eight final variables we determined to be most predictive of treatment seeking status, four of those variables were subscales of the WISDM-68. As seen in Figure 3, the effects for these variables were very small. For social-environmental goads, there was a general negative trend in which log odds of seeking treatment decreased as social-environmental goads increased. When examining loss of control, there was a general positive trend such that as loss of control increased, the log odds of treatment seeking increased. Lastly, craving and tolerance showed similar results such that there was a relatively stable negative log odds of seeking treatment regardless of scores on these two WISDM-68 subscales.

## Discussion

Treatment seeking for smoking cessation has tremendous clinical and health implications with the potential to reduce tobacco-related morbidity and mortality. Heavy drinking smokers constitute a distinct subset of smokers who are more likely to experience alcohol-induced smoking lapses during a quit attempt (9, 10). The negative impact of the co-use of cigarettes and alcohol has been well documented across a range of health outcomes. However, determinants of treatment seeking for smoking cessation in this important subgroup of smokers remain understudied.

This study used a data driven approach with groups of heavy drinking smokers who were either treatment-seeking or non-treatment seeking for smoking cessation to test clinical variables associated with the likelihood of “belonging” to the treatment-seeking group. Notably, our collective sample consisted of heavy drinking smokers from two separate research studies, one targeting those who were interested in treatment and one that was not recruiting those interested in treatment. Results revealed that age and drinks per drinking day were among the most robust predictors such that younger individuals were less likely to seek treatment for smoking cessation. Conversely, individuals at lower drinks per drinking day were more likely to belong to the treatment seeking group. These results suggest that in addition to having a negative impact on the success of a smoking quit attempt, heavy drinking may have an adverse impact on the likelihood of seeking treatment for smoking cessation. In addition, cigarettes per smoking day was consistently negatively associated with treatment seeking. Regarding age, it may be that those in a younger age range are less likely to seek treatment and that additional efforts are needed to engage this group in smoking cessation efforts. Older adults are more likely to be chronic smokers at risk for health problems related to their smoking (33, 34). Therefore, older individuals might have greater motivation to seek treatment because of the health benefit and threat of negative consequences from failure to successfully quit smoking being more salient (i.e., more immediate). Demographic and contextual factors such as education, marital status, and number of smokers in one’s social network have been associated with initial abstinence rates and risk for smoking lapse (35). Different life stages may give way to change in priorities for these various contextual factors that could make it more difficult to prioritize treatment. Lastly, greater cognitive impulsivity was also indicative of lower log odds of seeking treatment. These results align broadly with the literature denoting the negative effects of alcohol on decision making among social drinkers (36) and cognitive impairments among younger adolescent heavy drinkers (37). Impulsive decision making may contribute to heavy drinking smokers being more likely to take risks related to their health by not seeking smoking cessation treatment.

A select few subscales from the WISDM-68 were found to have an impact, albeit small, on the likelihood of belonging to the treatment seeking group. The positive association between loss of control and treatment seeking the compulsive nature of one’s cigarette use may lead to contemplation of changing their smoking behavior, however, not yet taking action to change their behavior as the log odds remained negative even at high values of loss of control. The negative association between social-environmental goads and belonging to the treatment seeking group underscores the importance of a social support network for engaging in smoking cessation, as previous research has made salient the impact social network can have on smoking cessation (38). The last two WISDM subscales implicated as top predictors were craving and tolerance. Regardless of severity of craving and tolerance, participants were less likely to seek treatment. A latent profile analysis of the 13 WISDM-68 subscales revealed a “primary” scale consisting of 4 subscales (automaticity, craving, loss of control, and tolerance) is a strong predictor of withdrawal, release, and other nicotine dependence criteria (39). This primary scale may reflect factors that are believed to underlie the pharmacological neuroadaptations that drive the automatic processes associated with cigarette use. In contrast, the “secondary” scale consisting of the remaining 9 subscales may reflect a variety of internal and external factors that impact dependence (39), however, are not pharmacologically driven factors impacting use (e.g., weight control, taste/sensory processes). Extending beyond these findings, in the present study, we found 3 subscales that are part of the “primary” scale are also associated with likelihood of belonging to the treatment-seeking group.

These findings should be interpreted in light of the study’s strengths and limitations. A primary strength of this study is the implementation of the random forest model, which compared to logistic regression or classification trees, results in less overfitting and increased robustness of findings. The large sample size and the clinical and demographic variables available provide adequate support for the data driven approach employed. Study limitations include the fact that the two samples were not randomly assigned and were not recruited concomitantly, although they were recruited using similar methods and drawing from the same community. Future observational studies examining predictors of treatment seeking status for smoking cessation among those who are both heavy drinkers and daily smokers would help generalize the preliminary findings herein.

Overall prediction accuracy of the model was somewhat limited and suggests that a host of other factors, not captured by our predictor variables, may have an impact on treatment seeking status. It is also important to note that degree of treatment-seeking status for smoking cessation may have varied among our treatment seeking sample, and impacted which variables were identified as top predictors in our sample. As data for the present study was pulled from the initial screening visit prior to randomization, it is possible a participant may have withdrawn from the study at a later time due to no longer wanting to engage in a formal quit attempt or take the smoking cessation medication. This may indicate less intent for seeking treatment that we could not distinguish at the time of the initial screening visit. Furthermore, it is possible that those who would self-identify as ‘treatment-seeking’ for participation in a research study may have varying degrees of intent to engage in treatment outside of a research study. Smoking cessation interventions have examined how treatment may be selected based on Prochaska’s 5 stages of change [precontemplation, contemplation, preparation, action, maintenance, (40–42)]. A Cochrane Review of stage-based changes for smoking cessation intervention found no conclusive evidence that stage-based interventions were more effective than non-stage-based interventions (43). We cannot definitively say that our treatment-seeking sample aligns with a specific stage of change. However, we can use the insights of what distinguishes those who self-select into the initial phases of a treatment-seeking versus non-treatment seeking research studies to understand factors impacting this initial attempt to receive some degree of treatment and promote continued efforts to reduce their smoking behavior outside of the discrete time spent within the study protocol. Future studies examining predictors of not only initial treatment-seeking behaviors by beginning to participate in a research study, but completion of quit attempt, success of quit attempt, and number of quit attempts before long-term cessation may also inform our understanding of important factors to consider for best aiding heavy drinking smokers in their smoking cessation efforts.

In closing, this study used data driven methods to identify variables associated with the likelihood of treatment seeking for smoking cessation among daily smokers who are also heavy drinkers. Results suggested that age and drinks per drinking day are two of the most robust predictors, followed by measures of nicotine craving and tolerance. These findings suggest that the negative impact of drinking on smoking cessation outcomes may extend to the treatment seeking process and that individuals who consume more drinks per drinking day are less likely to belong to the treatment-seeking group, compared to those who drink less. Implications of these results suggest that targeting heavy drinking smokers, particularly younger individuals, may be necessary to engage this group in smoking cessation efforts.

## Data availability statement

The data analyzed in this study is subject to the following licenses/restrictions: Existing datasets analyzed for this article are not publicly available. Requests to access these datasets should be directed to LR.

## Ethics statement

The studies involving human participants were reviewed and approved by Institutional Review Board of the University of California, Los Angeles. The patients/participants provided their written informed consent to participate in this study.

## Author contributions

LR designed the studies. JL, HR, and RG conducted the data analyses. RG, JL, and LR drafted the manuscript. All authors contributed to the interpretation of the data, revised the manuscript, provided their approval of the current version submitted for publication, agree to be accountable for all aspects of the work, and including its accuracy and integrity.
